# Targeting complement hyperactivation: a novel therapeutic approach for severe pneumonia induced by influenza virus/staphylococcus aureus coinfection

**DOI:** 10.1038/s41392-023-01714-y

**Published:** 2023-12-29

**Authors:** Leili Jia, Haihua Luo, Lizhong Li, Mingyao Wang, Jiangfeng Liu, Yuan Liang, Shan Li, Yong Jiang, Juntao Yang, Hongbin Song

**Affiliations:** 1 Institute for Disease Control and Prevention of PLA, Beijing, China; 2 Guangdong Provincial Key Laboratory of Proteomics, State Key Laboratory of Organ Failure Research, School of Basic Medical Sciences, Southern Medical University, Guangzhou, 510515 China; 3 State Key Laboratory of Common Mechanism Research for Major Diseases, Institute of Basic Medical Sciences, Chinese Academy of Medical Science and Peking Union Medical College, 100005 Beijing, China

**Keywords:** Infectious diseases, Target identification

**Dear Editor**,

Infections by the influenza virus are a significant and widespread global health threat, as these infections have an annual death toll ranging from 290,000 to 650,000.^1^ A significant proportion of these fatalities are attributed to secondary bacterial pneumonia, a severe complication commonly caused by ubiquitous respiratory pathogens such as *Staphylococcus aureus* (*S. aureus*).^2^ Of particular concern is the increased morbidity and mortality rates in individuals infected simultaneously with influenza virus and methicillin-resistant *S. aureus* (MRSA).^3^ The concurrent presence of bacteria and influenza virus usually causes acute respiratory distress syndrome (ARDS), which is associated with acute lung injury (ALI), severe lung tissue edema, and widespread inflammation. Nevertheless, determining the complex mechanisms underlying the synergistic interplay will require further investigation with a suitable coinfection mouse model. In our previous study, we utilized different sequential coinfections at various time points to model influenza A virus and MRSA coinfection. We found that mice infected with influenza A virus (H1N1A/Puerto Rico/8/34, also known as PR8, at a concentration of 1 × 10^2^ TCID50/ml) followed by exposure to MRSA (5 × 10^7^ CFU/ml) after 2 days (referred to as the d-2 group, Supplementary Table S1) experienced significant mortality and lung tissue damage. Significantly, the d-2 group displayed the highest mortality rate and the most severe lung damage on the fifth day of coinfection.^4^ Moreover, the increasing viral loads (Supplementary Fig. S1) hinted at a connection between lung damage and inflammatory cell infiltration, suggesting that the high mortality rate after H1N1A/MRSA coinfection may be due to overactivation of the inflammatory response, although validating this hypothesis will require further systematic investigation.

Based on these results, we performed single-cell RNA sequencing (scRNA-seq) analysis to investigate the pathogenesis of sequential H1N1A/MRSA coinfection and to study the multimodality of the immune response in the lung (Fig. 1a). We generated lung atlases by mapping 196,918 cells from mouse tissues using the following five infection conditions: PR8 alone, MRSA alone, and coinfection for 24/72/120 h. Nineteen cell types with significantly altered gene expression profiles were identified using downscaling and uniform manifold approximation and projection (UMAP) data visualization (Fig. 1b and Supplementary Fig. S2). As communication between different cell types is involved in immune regulation, CellChat was used to investigate cell‒cell interactions in different infection conditions. After pathogen infection, neutrophils rapidly migrate to the infection site and contribute to acute inflammation. Therefore, as we expected, sequential H1N1A/MRSA infection led to increased communication between neutrophils and other cell types (Supplementary Fig. S3). Moreover, the increasing viral loads (Supplementary Fig. S1) hinted at a connection between lung damage and inflammatory cell infiltration, suggesting that the high mortality rate after H1N1A/MRSA coinfection may be due to overactivation of the inflammatory response; however, confirming this hypothesis will require further systematic investigation.

**Fig. 1:**
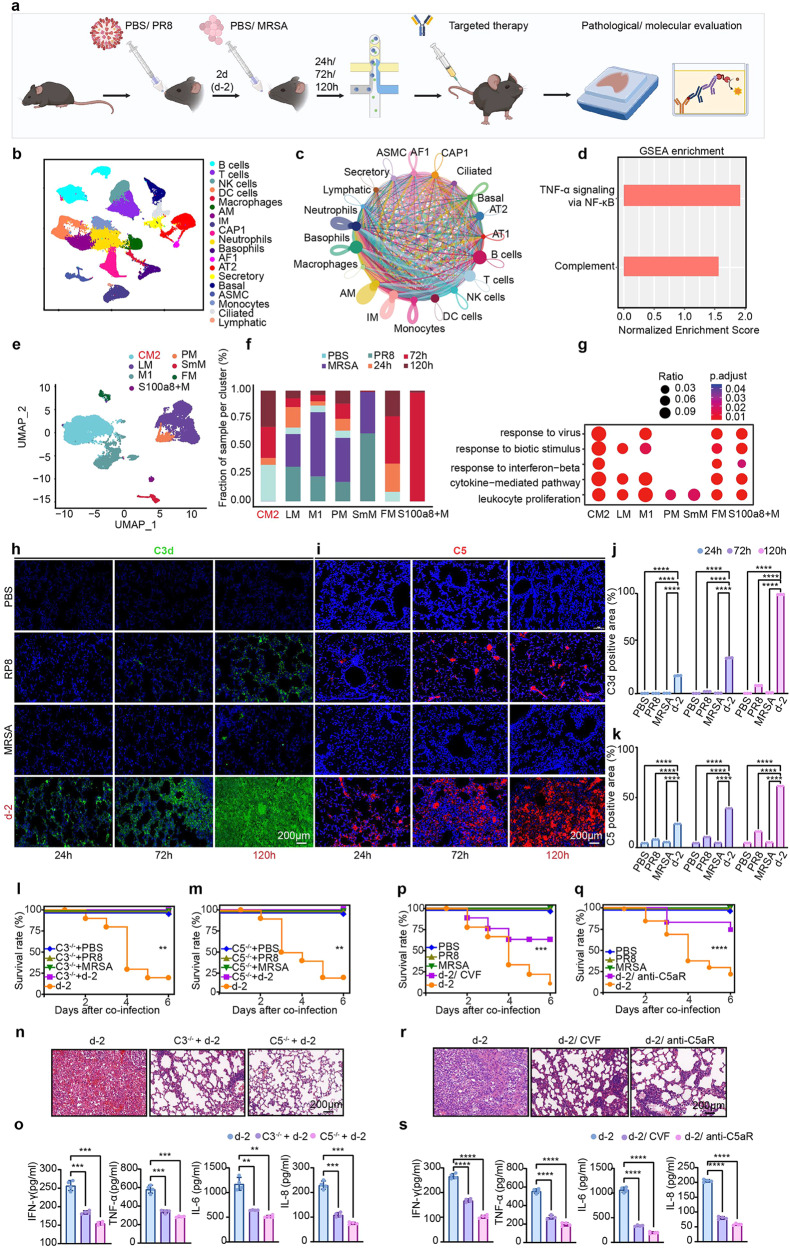
Inhibiting complement over-activation demonstrates therapeutic efficacy in H1N1A/MRSA co-infected mice. **a** Schematic illustration of developing H1N1A/MRSA co-infected mouse model and treatment strategies. **b** UMAP plot of 196,918 cells from mice treated with PBS, MRSA, PR8, and coinfection for 24 h/72 h/120 h. **c** Differential interaction strength of significant ligand-receptor pairs for all cell clusters. **d** Top pathways from GSEA of T cells. **e** UMAP plot of seven subsets of macrophages colored by cluster identification. **f** Ratio of all cells belonging to the macrophages clusters. **g** Bubble plot showing the expression of functional pathways of macrophages in different groups. Immunohistochemistry of lung tissues was performed to detect the accumulation of complement (**h**) C3d and (**i**) C5 in co-infected mice with different treatments over time. The scale bar represents 200 μm. Histogram of the percentage of (**j**) C3d and (**k**) C5 positive area. *N* = 3/group. The survival curve of (**l**) C3 knock-out (C3^−/−^), (**m**) C5 knock-out (C5^−/−^). **n** Hematoxylin and eosin (H&E) staining in representative mouse lung sections among knock-out groups (d-2 coinfection, C3^−/−^ and C5^−/−^ treated groups). **o** Quantification of serum IFN-γ, TNF-α, IL-6 and IL-8 levels by ELISA. *N* = 4/group. The survival curve of (**p**) CVF treated and (**q**) anti-C5aR treated mice. **r** H&E staining in representative mouse lung sections among therapy groups (d-2 coinfection, CVF treated and anti-C5aR treated groups). **s** Quantification of serum IFN-γ, TNF-α, IL-6 and IL-8 levels by ELISA. *N* = 4/group. All quantitative results were presented as mean ± SD. Type Il pneumocyte, T2P, inflammatory pneumocyte, IP; type I pneumocyte, T1P; lfitm3+ pneumocyte, lfitm3+P; B-cell-stimulating pneumocyte, BP. All the experimental groups in (**g**, **h**) were compared by a two-way ANOVA followed by Bonferroni’s multiple comparisons test; *p* < 0.05 indicates a significant difference. Ns, *p* > 0.05; *p < 0.05; **p < 0.01; ***p < 0.001; ****p < 0.0001. All the experimental groups in (**n**) and (**p**) were compared by a one-way ANOVA followed by Benjamini and Hochberg multiple comparisons test; *p* < 0.05 indicates a significant difference. Ns, *p* > 0.05; *p < 0.05; **p < 0.01; ***p < 0.001; ****p < 0.0001

Disruption of the alveolar barrier and the dysregulation of inflammation are key pathophysiological factors in coinfection-induced acute lung injury and acute respiratory distress syndrome. As increased permeability resulting from dysfunction of the alveolar barrier is a defining characteristic of ALI in both clinical and experimental settings, our initial focus was a detailed analysis of subtypes of alveolar epithelial cells. We observed a markedly higher percentage of inflammatory and interferon-induced transmembrane protein 3 (Ifitm3+) pneumocytes, along with an increase in inflammatory alveolar epithelial cells, in the coinfected group than in the control group (Supplementary Figs. S4a and S5a–c). The functionalities of these subtypes of alveolar epithelial cells were closely linked to inflammatory responses, such as the response of IFN-γ (Supplementary Fig. S5d). This result strongly implies that disruption of the immune system and inflammation resulted in alterations to different types of cells during coinfection. Furthermore, a crucial factor in resolving ALI is the effective removal of neutrophils from the alveolar lumen. Therefore, identifying targets for intervention in coinfection depends on clarifying the process of neutrophil clearance. The uptake of neutrophils by alveolar macrophages through phagocytosis is an important pathway of neutrophil removal. Our analysis of macrophage subtypes has shown that M2 macrophages dependent on complement clusters increase complement activation, as evidenced by the high expression levels of complement activation marker genes such as chemokine ligand 2 (Ccl2) and genes in pathways associated with complement activation, such as the IFN pathway (Supplementary Fig. S4b and Fig. 1e–g). These results imply that complement activity may be overactivated during coinfection. Additionally, T cells may expedite neutrophil clearance. The findings of gene set enrichment analysis (GSEA) (Fig. 1d) revealed that coinfection promoted proinflammatory responses linked to the complement system in T cells, such as tumor necrosis factor α (TNF-α) signaling conveyed by nuclear transcription factor κ B (NF-kB), as well as complement processes (Fig. 1d). Overall, coinfection with H1N1A/MRSA resulted in excessive activation of the complement system, which involves numerous cellular components. Addressing the overactivation of the complement system could serve as a promising therapeutic option for coinfection.

To confirm the involvement of complement activation in coinfection-induced acute lung injury, fluorescence staining for C3 deposition (C3d) and C5 was performed on lung tissue obtained under different infection conditions. Consistent with our previous histology results,^4^ the d-2 coinfection group, which had the most severe lung damage, showed the highest levels of C3 and C5 deposition, in contrast to that seen in the single pathogen infection groups (Fig. 1h–k). Further research was conducted on C3 and C5 mutant mice (C3^−/−^ and C5^−/−^) that lack the complement protein C3 or C5 to understand the underlying mechanisms. C3- or C5-deficient mice coinfected with H1N1A/MRSA had a significant reduced mortality rate compared to coinfected wild-type mice (Fig. 1l, m). These knockout mice also had reduced lung inflammation, suggesting better recovery from pathological damage (Fig. 1n). To further elucidate whether this reduction was due to an alleviated inflammatory response, the levels of proinflammatory factors (IFN-γ, TNF-α, interleukin 6 [IL-6] and interleukin 8 [IL-8]) in bronchoalveolar lavage fluid (BALF) were assessed. The results showed that coinfection caused an increase in the levels of proinflammatory factors, while C3 or C5 knockout caused a significant reduction (Fig. 1o). These results showed that decreases in the level of complement molecules led to a significant reduction in mortality, lung injury, and immune disorder, suggesting that inhibition of complement activation may suppress the development of ARDS with H1N1A and MRSA coinfection.

Thus, further research was conducted to verify the benefits of inhibiting complement activation through complement-targeted therapies. Cobra venom factor (CVF), which targets C3d, and anti-C5aR monoclonal antibodies were assessed for their therapeutic potential in a coinfection model. Previous studies have shown that CVF can efficiently diminish the levels of complement in various animal models; thus, the therapeutic efficacy of CVF for H1N1A/MRSA coinfection was evaluated. The results showed that mice treated with CVF had lower mortality rates (Fig. 1p) and less pathological damage than the d-2 group without treatment (Fig. 1r). As expected, mice treated with CVF also had lower levels of proinflammatory factors (Fig. 1s). Meanwhile, anti-C5aR monoclonal antibodies, which are widely used in targeted complement treatments in clinical settings, were also effective against coinfection when administered intravenously through the caudal vein. Anti-C5aR treatment significantly increased survival rates (Fig. 1q) and substantially decreased inflammatory infiltration and the release of proinflammatory factors (Fig. 1r, s). We also explored the effectiveness of targeting factors that regulate the complement system, with supporting details provided in the Supplementary Materials (Supplementary Fig. S6). Overall, these findings suggest that therapies targeting the complement system could be effective in treating severe H1N1A/MRSA coinfection.

Influenza virus infection followed by secondary bacterial invasion is a known causative factor that promotes severe lung injury and inflammation.^5^ As such, there is an exigency to uncover the underlying pathogenesis and to develop effective therapies. Our study showed that complement pathways were activated in mice coinfected with H1N1A/MRSA, which led to ALI and increased mortality. Moreover, new therapeutic approaches have been proposed to address this issue, which effectively inhibit complement activation by targeting C3 or C5 using CVF or anti-C5aR, respectively. The targeting strategy efficiently reduced complement overactivation, leading to decreased lung injury and mortality. Taken together, these findings suggest that complements could be the key mediators of coinfection, and targeting complements can be an innovative clinical treatment strategy for managing coinfections with viruses and bacteria.

### Supplementary information

Supplementary materials

Supplementary Table S1

## Data Availability

I am pleased to inform you that our data have been released and are currently in a publicly accessible status on the China National Center for Bioinformation (CNCB) website. Here is the direct link for quick access: https://ngdc.cncb.ac.cn/gsa/browse/CRA013573.
