# Supplementary material for: Targeting complement hyperactivation: a novel therapeutic approach for severe pneumonia induced by influenza virus/staphylococcus aureus coinfection
Source: Signal Transduct Target Ther. 2023 Dec 29;8:467. doi: 10.1038/s41392-023-01714-y (PMC10754916; doi:10.1038/s41392-023-01714-y)
Supplement: Supplementary file 1 — Supplementary materials [file 41392_2023_1714_MOESM1_ESM.docx]

Supplemental Materials for

Targeting Complement Hyperactivation: A Novel Therapeutic Approach for Severe Pneumonia Induced by *Influenza Virus/Staphylococcus Aureus* Coinfection

Leili Jia^1Δ^, Haihua Luo^3Δ^,Lizhong Li^1Δ^, Mingyao Wang^2Δ^, Jiangfeng Liu^2Δ^, Yuan Liang^1^, Shan Li ^3^, Yong Jiang^3^*, Juntao Yang^2^*, Hongbin Song^1^*

Correspondence to: [hongbinsong@263.net](mailto:hongbinsong@263.net), [yangjt@pumc.edu.cn](mailto:yangjt@pumc.edu.cn), jiang48231@163.com

**This PDF file includes:**

Supplementary method

Supplementary figure S1-6

**Supplementary Method**

**Mice**

Six-week-old C57BL/6 (B6) mice were purchased from Beijing Vital Lihua Experimental Animal Center. C3^−/−^ (C57/B6), C5^−/−^ (BALB/c), and C3aR^−/−^ (BALB/c) deficient mice were obtained from the Jackson Laboratory (USA). All mice were housed in the Specific pathogen Free (SPF) environment of the Laboratory Animal Center of Academy of Military Medical Sciences of China. All experiments were performed in accordance with the relevant institutional animal care and use guidelines.

**Viral Strains and Bacteria**

Influenza strain A/Puerto Rico/8/34 (H1N1) of influenza A virus (PR8) was from our laboratory. After propagation in 9-d-old chicken embryos, the virus stock was titrated and stored. Methicillin-resistant *Staphylococcus aureus* (MRSA) was isolated and stored in our laboratory.

The details of MRSA strain isolation:

(1) Take an MRSA slant strain, sterile pluck a ring of moss, inoculate with LB culture solution and leave to rest. Incubate for 18 hours as a seed liquid.

(2) Contains 5 ml sterile LB in vitro culture of eight, numbered 0, 1, 2, 3, 4,5, 6, 7 h.

(3) 50µL of seed liquid is collected and added to 8 numbered LB test tubes at 37℃ and 200 rpm. The OD value was measured at the appropriate time and 15µL of Kana was removed at 50µg/mL. The plate was coated with resistant LB solid medium.

(4)The OD value was determined by ultraviolet spectrophotometer ND-1000, and the LB culture plate was cultured at 37℃ overnight. The colonies were then counted and the CFU values were calculated.

(5)5x107 CFU/mL MRSA was applied for further research

**Infections and groups**

Mice were deeply anesthetized by 40 μL/10 g body weight standard injection of 2% sodium pentobarbital (Pentobarbital Sodium), followed by intranasal infection with influenza virus and bacteria. Viral inoculation dose and volume: A, the non-lethal dose PR8 was 10^0.5^ TCID50/mL of influenza virus with the volume adjusted to 25 μL with PBS; B, the lethal dose of PR8 was 10^4.5^ TCID50/mL of influenza virus with the volume adjusted to 25 μL with PBS. Bacterial inoculation dose and volume: A, the non-lethal dose of MRSA was 5×10^7^ CFU/mL MRSA with the volume adjusted to 40 μL with PBS; B, the lethal dose of MRSA was 40×10^7^ CFU/mL MRSA with the volume adjusted to 40 μL with PBS. Co-infected mice were established using a non-lethal dose of influenza virus and MRSA, and mouse mortality and body weight changes were observed daily. The grouping of mice is shown in Table 1.

**ELISA determination of C3a, C5a, TCC, and three pathways of complement activation.**

The lungs of mice were removed and placed in a 2 mL EP tube containing 1 mL of PBS and 1% protease inhibitor, and then automatically ground in a high-throughput tissue grinder. The supernatant was collected by centrifugation at 10,000 ×g for 15 min at 4°C. The contents of C3a, C5a, and TCC were detected by an ELISA kit (Genemei). The complement consumption of the three pathways was examined using the complement pathway kit (Wiselab). The experimental method followed the kit instructions.

**Histology**

Fresh lung tissue was fixed with 4% paraformaldehyde for more than 24 hours. The lung tissue was embedded in paraffin and sectioned. The pathological score was determined using a blind randomized method. The specific evaluation indicators were as follows: 0 = no airway necrosis, 1 = focal necrosis, 2 = some confluence of necrosis in larger airways, and 3 = confluent airway necrosis in most airways.

**Measurement of complement deposition**

Lung tissue sections were incubated with anti-mouse C3d fluorescein iso-thiocyanate (FITC) (R&D, AF2655) antibody for 1 hour at room temperature, and then the nuclei were counterstained with DAPI (Thermo Scientific, Rockford, IL). The fluorescence level was then determined using a Zeiss LSM5 Confocal microscope. A score of 0–3 represented different concentrations as follows: 0 = no staining, 1 = mild, 2 = moderate, and 3 = intense.

**Inhibition of complement activation with C3d-ScFv-CD59 attenuates lung injury induced by influenza/bacterial co-infection**

In this study, C3d-ScFv was used as a target molecule and CD59 as effecter molecules. C3d-ScFv-CD59 targeted complement inhibitors were generated to investigate a new strategy for the treatment of severe pneumonia caused by influenza/bacterial co-infection in mice. Human recombinant C3d protein and a large capacity fully synthesized human phage single chain antibody library were used. After three rounds of screening by phage display technology, three strains of human phage single chain antibody A1, A3, and B6 with relative molecular weights of about 25 KDa were obtained. The equilibrium dissociation constant of B6 was the lowest and its binding ability to C3d molecules was the best; therefore, it was the first choice for the construction of a single chain antibody in the targeted complement inhibitor. The targeted complement inhibitors, C3d-ScFv-CD59, were amplified and expressed in accordance with their expected sizes. The binding force of the two targeted complement inhibitors and C3d reached the nanomolar level, and they were close to the equilibrium dissociation constant (KD) for C3d-ScFv with high affinity. Within the concentration ranges of 0.0–1.5 µmol/l and 0.0–24 µmol/l, respectively, C3d-ScFv-CD59 had a higher inhibition rate and inhibitory effect than the corresponding individual effector molecules CD59. The inhibitory effect of complement in C3d-ScFv-CD59 was negatively correlated with the concentration of the binding C3d molecules, indicating that C3d components in the complement activated region were identified by C3d-ScFv-CD59 and had a role in the complement inhibitory effect. Under the existing natural environmental conditions, the mouse model of influenza/bacterial co-infection was successfully induced, and co-infected mice were treated with different doses of C3d-ScFv-CD59, respectively. The survival rate was significantly improved, with the highest survival rate at 15µg/mouse and 60µg/mouse, respectively. Compared with the single effect molecules CD59 treatment groups and the blank control group, mice treated with C3d-ScFv-CD59 had significantly higher survival rates, significantly reduced lung pathological injury and levels of complement activation product C3d. Furthermore, the targeted complement inhibitors were concentrated in the lung lesion areas and the lung index was significantly decreased.

**Statistical Analysis**

Statistical analysis was performed using GraphPad Prism 5, multiple sets of comparisons using one-way analysis of variance (ANOVA) and Benjamini and Hochberg's post hoc test or two-way ANOVA followed by Bonferroni’s multiple comparisons test, which depends on the experimental design. And experimental data expressed as mean ± standard error (SEM). *p <*0.05 indicates significant difference. Ns, p>0.05; *, p<0.05; **, p<0.01; ***, p<0.001; **** p<0.0001.

**Primary analysis of raw scRNA data**

The raw sequence data reported in this paper have been deposited in the Genome Sequence Archive (Genomics, Proteomics & Bioinformatics 2021) in National Genomics Data Center (Nucleic Acids Res 2022), China National Center for Bioinformation / Beijing Institute of Genomics, Chinese Academy of Sciences that are publicly accessible at <https://ngdc.cncb.ac.cn/gsa> (GSA: CRA013573)^1,2^. Raw reads from scRNA-seq were processed to generate gene expression matrix using CeleScope (https://github.com/singleron-RD/CeleScope) v1.10.0 pipeline. Briefly, raw reads were first processed with CeleScope to remove low quality reads with Cutadapt v1.17^3^ to trim poly-A tail and adapter sequences. Cell barcode and UMI were extracted. After that, we used STAR v2.6.1b^4^ to map reads to a custom reference genome encompassing murine GRCm38 (ensembl version 102 annotation)、influenza virus A/Puerto Rico/8/1934(H1N1)(PR8)(NCBI: txid211044)、 Methicillin-resistant Staphylococcus aureus (MRSA). UMI counts and gene counts of each cell were acquired with featureCounts v2.0.1^5^ software, and used to generate expression matrix files for subsequent analysis. And a total of 196,918 cells are identified.

**Quality control, dimension-reduction and clustering**

Barcodes with < 200 unique genes, <1,000 UMI counts, and > 10% of transcript counts derived from mitochondrially encoded genes were discarded. We used functions from Seurat v4.1.1^6^ for dimension-reduction and clustering. Data was normalized and scaled using the SCTransform function. To identify major axes of variation within our data, we first examined only highly variable genes across all cells, yielding approximately 3,000 variable genes. An approximate principal component analysis was applied to the cells to generate 100 principal components (PCs). Using a combination of the Jackstraw function in Seurat and observing the ‘‘elbow’’ of the standard deviations of PCs, we chose the top 50 PCs for subsequent clustering and visualization. Doublets were detected, marked & removed elaborately for each sample with DoubletFinder (v2.0.3), and finally 134,238 cells were obtained for downstream analysis. Batch effect between 18 samples was removed by Harmony v0.1.0^7^. To identify clusters of transcriptionally similar cells, we employed unsupervised clustering as described above using the FindClusters tool within the Seurat R package with default parameters and resolution set to 0.3. Finally, UMAP algorithm was applied to visualize cells in a two-dimensional space. Finally, 22 clusters were clustered from 18 samples.

**Differentially expressed genes (DEGs) analysis**

To identify differentially expressed genes (DEGs), we used the Seurat FindMarkers function based on Model-based Analysis of Single-cell Transcriptomics (MAST) with default parameters, and selected the genes Bonferroni-adjusted p value cutoff < 0.05 and with an average log (Fold Change) value greater than 0.25 as DEGs.

**Cell type annotation**

The cell type identity of each cluster was determined with the expression of canonical markers found in the DEGs using local database and cell marker available on LungMap、 SingleR (v 1.8.1) and in the published literature. dot plots/violin plots displaying the expression of markers used to identify each cell type were generated by Seurat v4.1.1 DotPlot/Vlnplot function. Finally, 19 cell types were annotated carefully.

**Pathway enrichment analysis**

To investigate the potential functions of DEGs, the Gene Ontology (GO) and Kyoto Encyclopedia of Genes and Genomes (KEGG) analysis were used with the “clusterProfiler” R package 4.2.2^8^. Pathways with p_adj value less than 0.05 were considered as significantly enriched. Gene Ontology gene sets including molecular function (MF), biological process (BP), and cellular component (CC) categories were used as reference.

For GSEA pathway enrichment analysis, the average gene expression of each cell type was used as input data using the fgsea(v1.20.0) package^9^. GSEA enrichment was conducted for each cell type and the corresponding feature gene sets were downloaded from mouse hallmark gene sets in MSigDB (Molecular Signatures Database) (<http://software.broadinstitute.org/gsea/msigdb>)

**References of Supplementary Method**

1 Chen, T. *et al.* The Genome Sequence Archive Family: Toward Explosive Data Growth and Diverse Data Types. *Genomics Proteomics Bioinformatics*. **19**, 578-583, (2021).

2 Database Resources of the National Genomics Data Center, China National Center for Bioinformation in 2022. *Nucleic Acids Res*. **50**, D27-d38, (2022).

3 Martin, M. Cutadapt removes adapter sequences from high-throughput sequencing reads. *EMBnet. journal*. **17**, 10-12, (2011).

4 Dobin, A. *et al.* STAR: ultrafast universal RNA-seq aligner. *Bioinformatics*. **29**, 15-21, (2013).

5 Liao, Y., Smyth, G. K. & Shi, W. featureCounts: an efficient general purpose program for assigning sequence reads to genomic features. *Bioinformatics*. **30**, 923-930, (2014).

6 Satija, R. *et al.* Spatial reconstruction of single-cell gene expression data. *Nature biotechnology*. **33**, 495-502, (2015).

7 Korsunsky, I. *et al.* Fast, sensitive and accurate integration of single-cell data with Harmony. *Nat Methods*. **16**, 1289-1296, (2019).

8 Yu, G., Wang, L.-G., Han, Y. & He, Q.-Y. clusterProfiler: an R package for comparing biological themes among gene clusters. *Omics: a journal of integrative biology*. **16**, 284-287, (2012).

9 Hänzelmann, S., Castelo, R. & Guinney, J. GSVA: gene set variation analysis for microarray and RNA-seq data. *BMC Bioinformatics*. **14**, 1-15, (2013).

Supplementary Figures


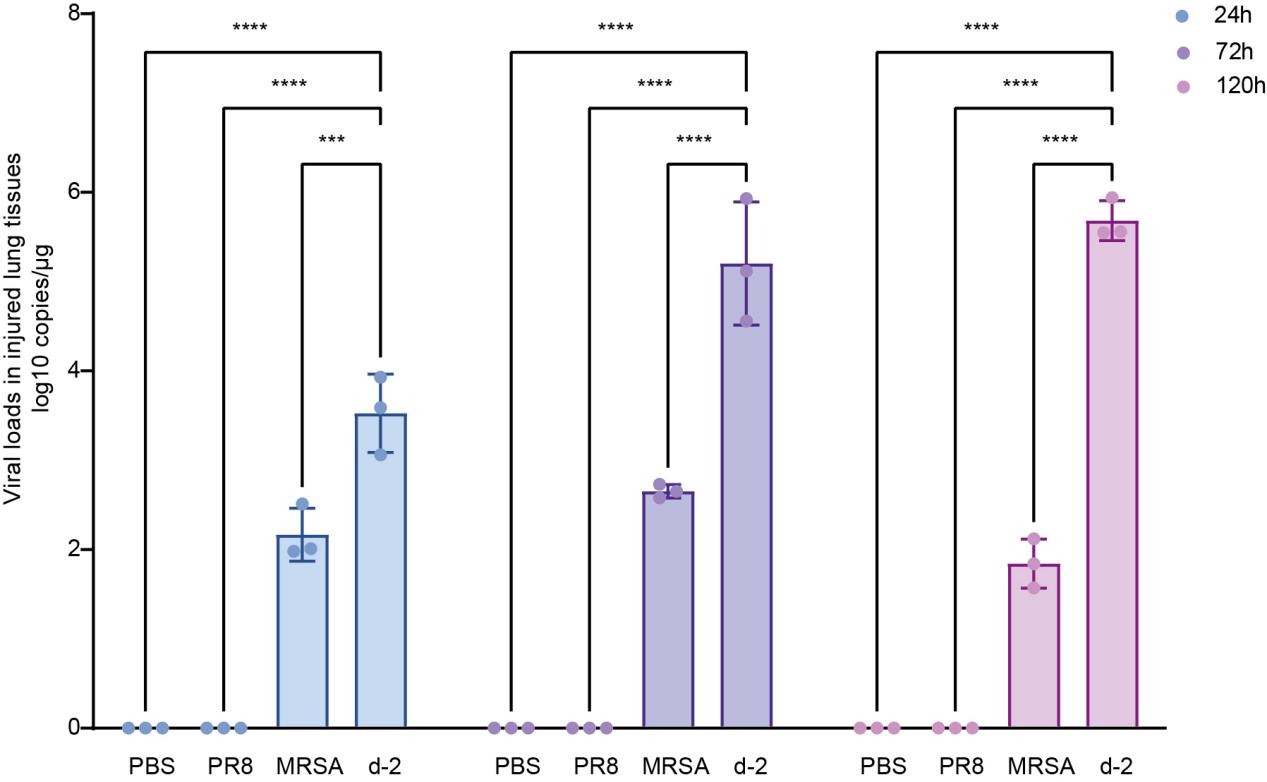


**Figure. S1 Bacterial burden of mice injured lung tissue infected with 1x10^2^ TCID50/mL PR8 and 5x10^7^ CFU/mL MRSA.**

All the experimental groups were compared by a two-way ANOVA followed by Bonferroni’s multiple comparisons test, n=3/group. Data were represented as mean ± SEM. NS, not significant; *p < 0.05, **p < 0.01, ***p < 0.001, ****p < 0.0001


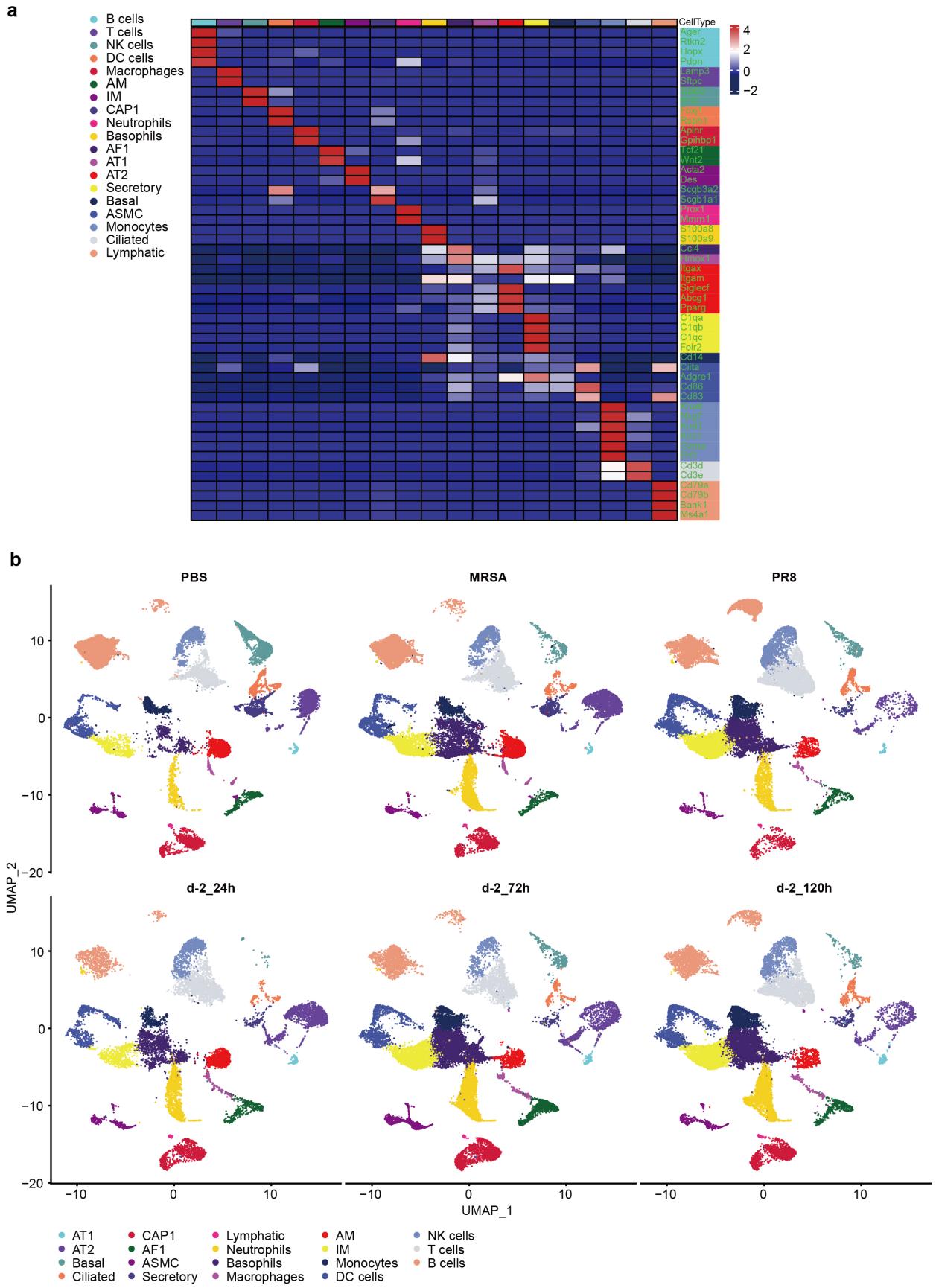


**Figure. S2 Uniform Manifold Approximation and Projection (UMAP) visualization of cells collected from co-infected mice lung.**

**(a)** Heatmap of the top transcripts in each cluster showed clear demarcation between different clusters. **(b)** Gene expression was measured simultaneously in single cell from lung homogenates after infection of co-infected mice with different infection sequences and times.


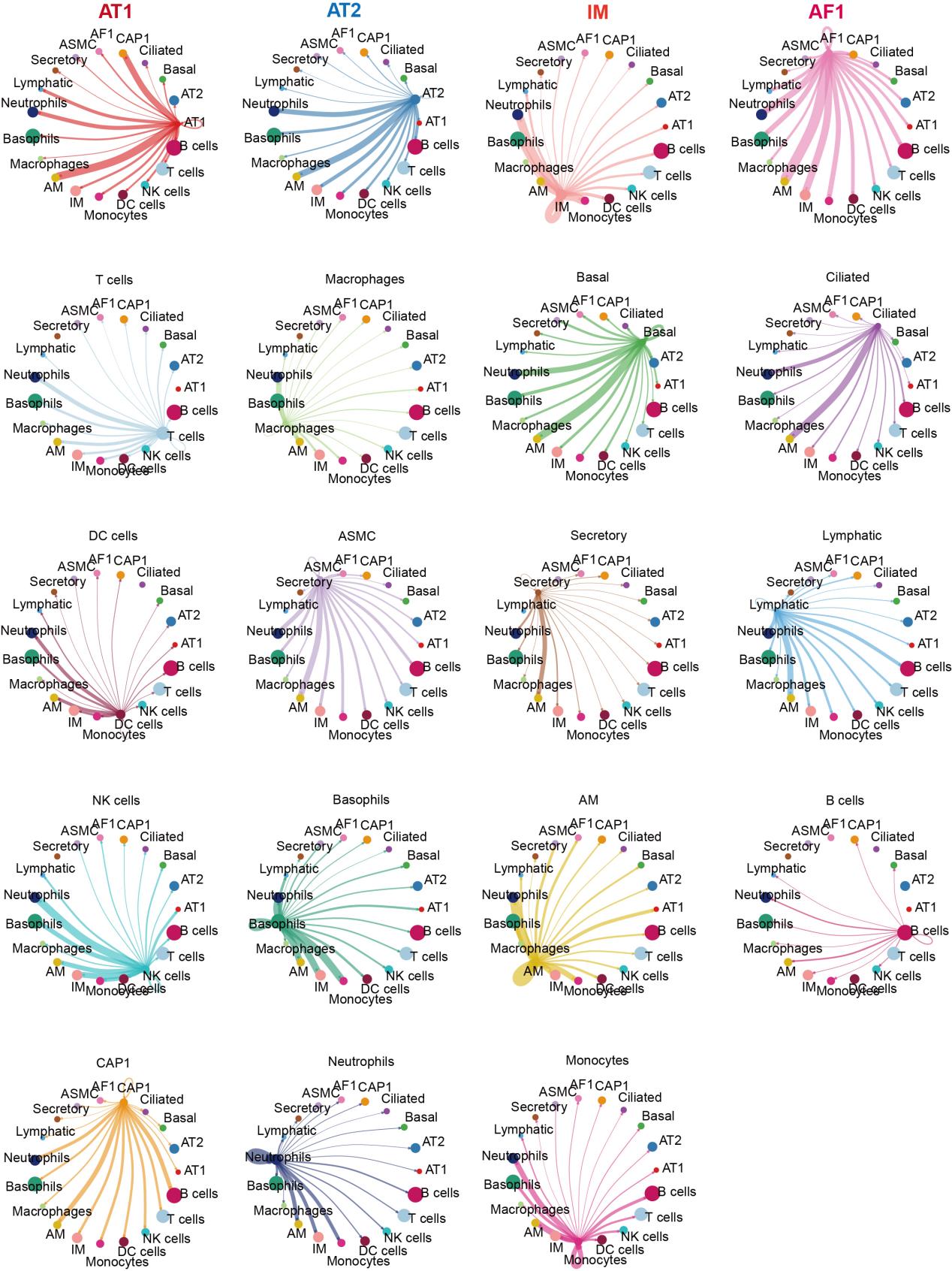


**Figure. S3 Cell interaction analysis of pulmonary cell clusters in treated mice.**

Detailed interaction strength of significant ligand-receptor pairs of other cell clusters.


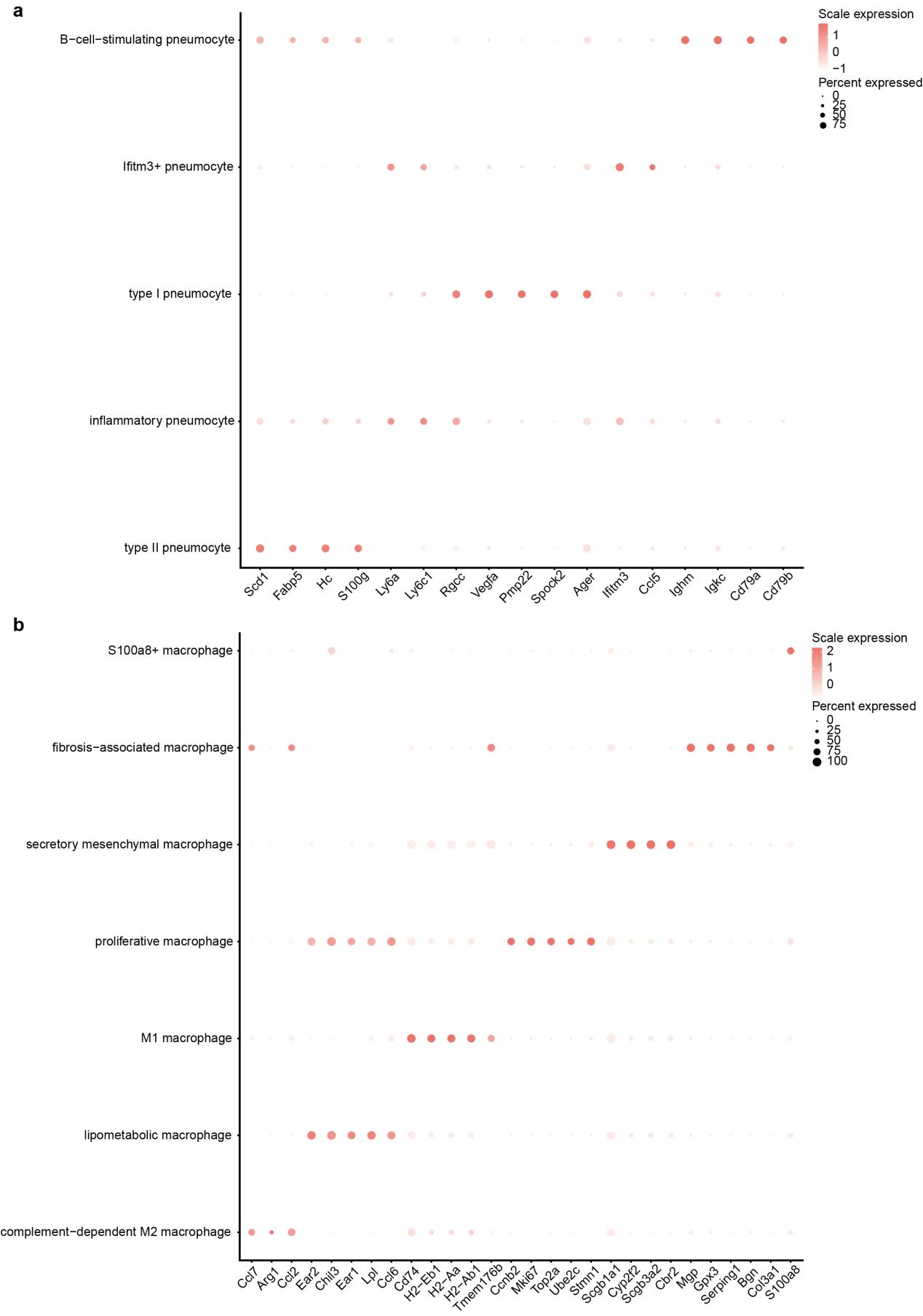


**Figure. S4 Dot plot showing the scale expression of selected signature genes for two groups based on subets of (A) pneumocytes and (B) macrophages.**

**
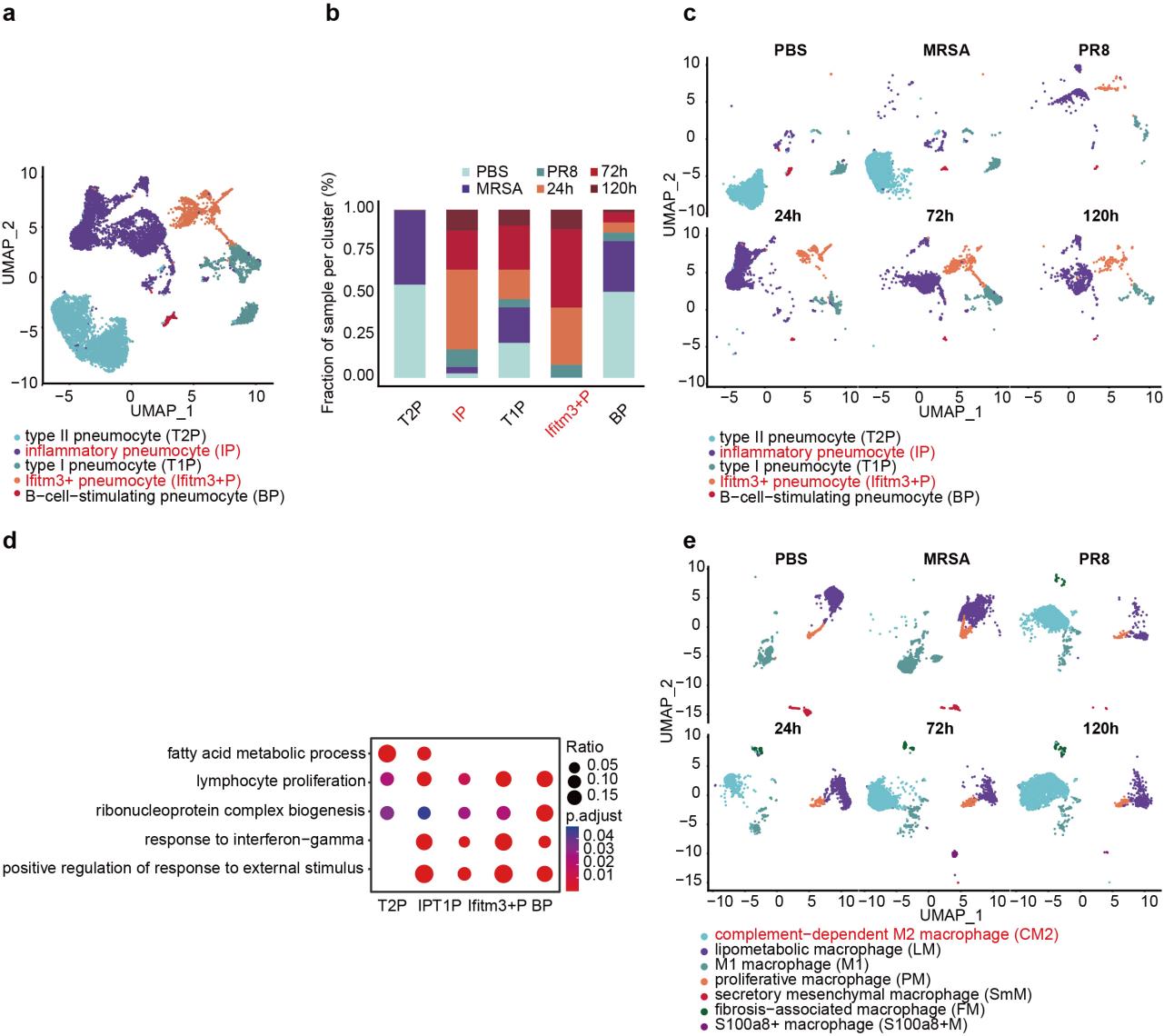
**

**Fig. S5 Co-infection motivates complement activation by mainly influencing pneumocytes and macrophages.**

UMAP plot of 5 subsets of pneumocyte colored by cluster identification. **(b)** Ratio of all cells belonging to the pneumocyte clusters. **(c)** UMAP plots showing the dynamic changes of pneumocyte clusters among each group. **(d)** Bubble plot showing the expression of functional pathways of pneumocytes in different groups. **(e)** UMAP plots showing the dynamic changes of macrophage clusters among each group.

**
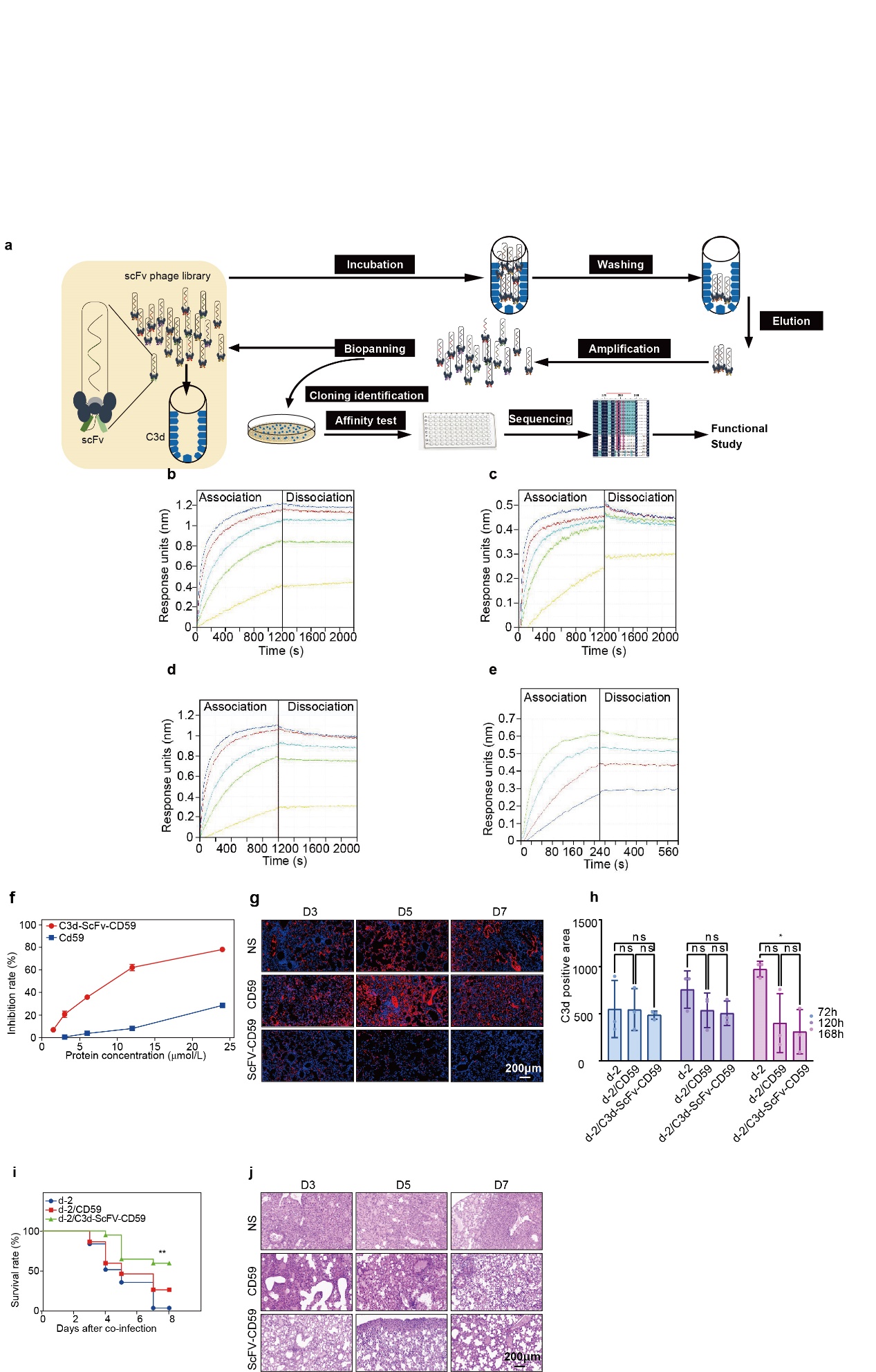
**

**Figure. S6 Construction and identification of targeted complement inhibitor C3d-ScFv-CD59**

1. Schematic illustration of developing and testing the targeted inhibitor. Binding and dissociation curves of single chain antibody **(b)** A1 and protein C3d, **(c)** A3 and protein C3d, **(d)** B6 and protein C3d, and **(e)** the targeted complement inhibitor C3d-ScFv-CD59 and protein C3d. **(f)** Inhibition of cell hemolysis is targeted by the complement inhibitor, C3d-ScFv-CD59, and the effector molecule, CD59. **(g)** Immunohistochemistry of lung tissues was performed to detect the accumulation of complement C3d in treated mice with different infection treatments over time. **(h)** Histogram of the percentage of C3d positive area. N=3/group. **(i)** The survival curve of treated mice. **(j)** H&E staining in representative mouse lung sections among treated groups. All quantitative results were presented as mean ± SD. All the experimental groups in panel g and h were compared by a two-way ANOVA followed by Bonferroni’s multiple comparisons test; p<0.05 indicates a significant difference. ****, *p*<0.00001, ***, *p*<0.0001, **, *p*<0.001, *, *p*<0.05, and NS indicates no significant difference.
