# Supplementary material for: Targeting complement hyperactivation: a novel therapeutic approach for severe pneumonia induced by influenza virus/staphylococcus aureus coinfection
Source: Signal Transduct Target Ther. 2023 Dec 29;8:467. doi: 10.1038/s41392-023-01714-y (PMC10754916; doi:10.1038/s41392-023-01714-y)
Supplement: Supplementary file 2 — Supplementary Table S1 [file 41392_2023_1714_MOESM2_ESM.docx]

Supplementary Table

Table S1: Group descriptions

| **Groups** | **Description** |
| --- | --- |
| **PBS** | **2x sham inoculation** |
| **MRSA** | **Only MRSA inoculation** |
| **PR8** | **Only PR8 inoculation** |
| d+0 (MRSA+PR8) | PR8 secondary inoculation 0 day after MRSA |
| d+1 (MRSA_1d+PR8) | PR8 secondary inoculation 1 day after MRSA |
| d+2 (MRSA_2d+PR8) | PR8 secondary inoculation 2 day after MRSA |
| d+3 (MRSA_3d+PR8) | PR8 secondary inoculation 3 day after MRSA |
| d-1 (PR8_1d+MRSA) | MRSA secondary inoculation 1 day after PR8 |
| **d-2 (PR8_2d+MRSA)** | **MRSA secondary inoculation 2 day after PR8** |
| d-3 (PR8_3d+MRSA) | MRSA secondary inoculation 3 day after PR8 |
| d-4 (PR8_4d+MRSA) | MRSA secondary inoculation 4 day after PR8 |
| d-5 (PR8_5d+MRSA) | MRSA secondary inoculation 5 day after PR8 |
| d-6 (PR8_6d+MRSA) | MRSA secondary inoculation 6 day after PR8 |
| d-7 (PR8_7d+MRSA) | MRSA secondary inoculation 7 day after PR8 |

‘d’ denotes *day*. MRSA, methicillin-resistant *Staphylococcus aureus*; PR8, influenza A virus/Puerto Rico/8/34
